# Refractory Fulminant Acute Disseminated Encephalomyelitis (ADEM) in an Adult

**DOI:** 10.3389/fneur.2014.00270

**Published:** 2014-12-23

**Authors:** Federico Rodríguez-Porcel, Alejandro Hornik, Jordan Rosenblum, Ewa Borys, José Biller

**Affiliations:** ^1^Department of Neurology, Loyola University Medical Center, Stritch School of Medicine, Maywood, IL, USA; ^2^Department of Radiology, Loyola University Medical Center, Stritch School of Medicine, Maywood, IL, USA; ^3^Department of Pathology, Loyola University Medical Center, Stritch School of Medicine, Maywood, IL, USA

**Keywords:** acute disseminated encephalomyelitis, corticosteroids, intravenous immunoglobulin, plasmapheresis, cyclophosphamide, rituximab

## Abstract

Acute disseminated encephalomyelitis (ADEM) is characterized by its rapid progression with variable symptoms and severity in adults and children. Multiple therapeutic options have been proposed, but solid evidence is yet to be gathered. We describe an adult man with a fulminant form of ADEM unresponsive to numerous treatment modalities.

## Case Presentation

A 60-year-old man was brought to the emergency room due to family concerns of rapid cognitive decline. Two weeks prior to hospitalization, he had an elective dental procedure. Immediately following the procedure, he complained of headaches, nausea, vomiting, intermittent fevers, and chills. He received one dose of intramuscular ceftriaxone, and daily oral amoxicillin and metronidazole. A week before admission to our hospital, he became progressively weaker, less communicative, and repeated non-sensical words.

Medical history was remarkable for arterial hypertension and dyslipidemia. Medications were simvastatin, amlodipine, and olmesartan.

Upon arrival to our hospital, he was afebrile, with normal blood pressure, heart rate, and oxygen saturation. He was alert and oriented only to person. Language was non-fluent. He was unable to follow commands, had moderate neck stiffness, moved all his extremities spontaneously, and responded appropriately to nociceptive stimuli.

Blood work showed hemoglobin (Hgb) of 12.5 g/dl, white blood cells (WBC) of 8.3 k/UL (85% granulocytes), and a platelet count of 263 k/UL. Basic metabolic profile (BMP) liver enzymes, albumin, bilirubin, and urine drug screen were unremarkable. Cranial computed tomography (CCT) was unremarkable. Empirical intravenous (IV) acyclovir 600 mg every 8 h, ceftriaxone 2 g every 12 h, vancomycin 1000 mg every 12 h, and metronidazole 500 mg every 6 h were started. A lumbar puncture (LP) showed an opening pressure of 7 cm of cerebrospinal fluid (CSF), 278 red blood cells (RBC), 84 WBC (88% lymphocytes), glucose content of 59 mg/dl, and a protein content of 91 mg/dl. CSF PCR for Herpes simplex virus (HSV) type 1 and 2, Epstein Barr virus (EBV), Cytomegalovirus (CMV), Enterovirus, Lyme antibodies, and cultures were negative. Antimicrobials were discontinued after a total of 7 days.

**Figure 1:**
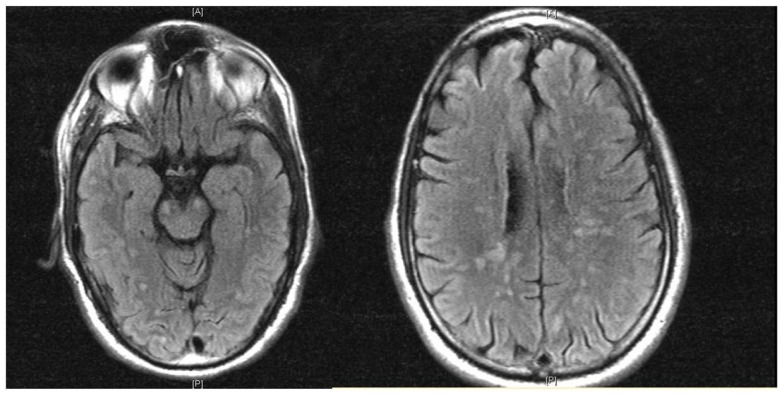
**MRI (FLAIR) shows multiple rounded lesions throughout the brain parenchyma predominantly involving the white matter of both cerebral hemispheres, basal ganglia and midbrain**.

**Figure 2:**
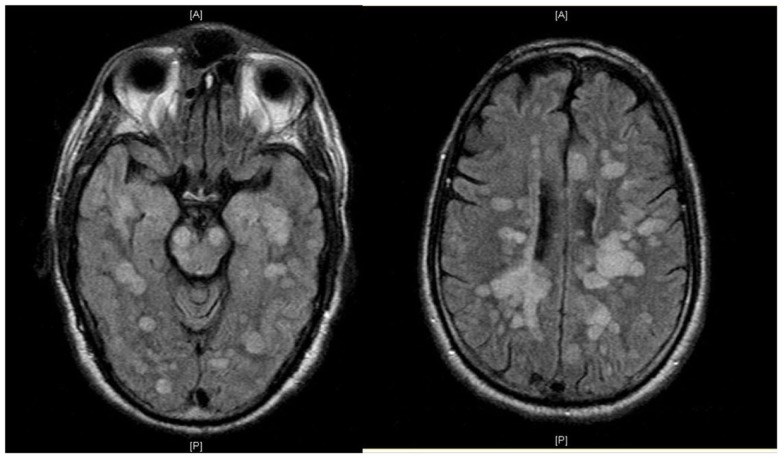
**MRI (FLAIR) shows increased number of supra and infratentorial lesions**.

**Figure 3:**
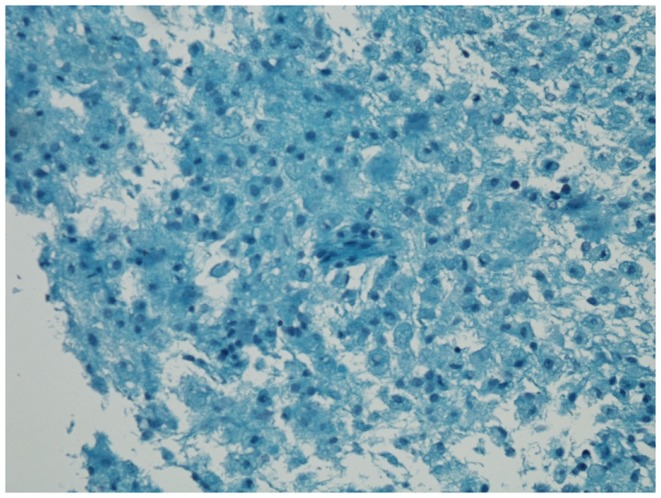
**Luxol Fast Blue stain shows areas of demyelination with relative sparing of the axons with some axonal swellings**.

He was admitted to the Neuroscience Intensive Care Unit (NICU). As his mental status further deteriorated, he was electively intubated for airway protection. EEG showed continuous spikes in the left frontal region. He received IV lorazepam 2 mg, and was started on IV levetiracetam 1000 mg every 12 h. He was placed on continuous video EEG monitoring for 48 h with no evidence of epileptiform activity. Magnetic resonance imaging (MRI) of the brain with gadolinium showed multiple rounded lesions throughout the brain parenchyma predominantly involving the white matter of both cerebral hemispheres, basal ganglia, midbrain, and pons. Some of these lesions showed enhancement in a linear and nodular pattern (Figure 1). Transesophageal echocardiogram (TEE) showed no sources of emboli or vegetations. Serial blood and urine cultures were negative. A presumptive diagnosis of ADEM was made, and he received methylprednisolone IV 1000 mg daily for 5 days and IV immunoglobulin (IVIG) 0.4 g/kg/dose daily for 5 days were concomitantly started. Due to the lack of improvement with IVIG, he received five sessions of plasma exchange (PLEX) every other day, starting the day after the last dose of IVIG. Despite this, the patient remained unresponsive to verbal stimuli, with preserved pupillary and oculocephalic reflexes and minimal arm flexion on noxious stimuli on the left arm. His exam remained unchanged during his hospital stay. Fourteen days after admission, a repeat MRI of the brain showed more disseminated white matter lesions (Figure 2). Stereotactic brain biopsy showed numerous cluster differentiation (CD) 68 positive cells with focal CD3 (T cell) and CD20 (B-cell) perivascular inflammatory infiltrates. The affected parenchymal areas contained both CD4 positive and CD8 positive T cells with the latter in greater number. Luxol Fast Blue stains showed demarcated areas of coalescencing perivenous demyelination with relative sparing of axons with some axonal swellings (Figure 3). Simian Virus (SV) 40 polyoma virus, Gram, and AFB stains were negative. Although the PAS stain without diastase was negative, the PAS with diastase stain showed questionable rod-shaped bacteria. He received another 6-day course sulfamethoxazole with trimethoprim 800–160 mg every 12 h. Subsequent bacterial PCR for tissue and serum were negative for *Tropheryma whipplei*. He then received IV Cyclophosphamide 750 mg/m^2^/dose. H. Subsequently, one dose of Rituximab 375 mg/m^2^/dose was given without significant change in his overall neurological condition. He was discharged after 30 days from the hospital to a long term acute care hospital where his condition remained unchanged, in a persistent vegetative state after 6 months, when this report was initially submitted.

## Literature Review

ADEM is a monophasic immune-mediated inflammatory disorder of the central nervous system (CNS) affecting predominantly the white matter of the brain, brainstem, and spinal cord. ADEM is more commonly seen in the pediatric population with a mean age of onset in this population of 4.5–7.5 years (1), and 33.5 years in adults (2). ADEM has been associated with numerous immunological triggers mainly viral infections or vaccinations, although in approximately one-third of cases in children and in approximately half of adults, ADEM showed no clear associations (3).

Diagnostic criteria for ADEM have been proposed (4), but the great overlap with the initial presentation of multiple sclerosis continues to present a challenge for the diagnosis without tissue confirmation.

Clinical presentation is usually characterized by acute onset encephalopathy associated with multifocal neurologic deficits. There is often a prodromal phase with fever, malaise, headaches, nausea, and vomiting, followed by meningismus. If an immunologic trigger is present, it usually precedes the presentation by 2–30 days (1). Presentation varies according to where the lesions are located. Compromise of cortical gray matter, basal ganglia (2), and bilateral optic nerve involvement are more frequent in ADEM compared to MS (5). Peripheral nervous system compromise can be present in up to 43% of the cases in adults (6).

Cerebrospinal fluid is usually normal or may show a mild lymphocytic pleocytosis, unlike MS. Oligoclonal bands (OCB) are present in 20–58% of adults compared to 85% in MS (2, 7). Some studies suggest that in ADEM, the presence of OCB is a transient phenomenon (6).

Magnetic resonance imaging in ADEM typically presents with large multiple and asymmetric lesions involving cortical, gray white matter junction, and central white matter as well as cerebellum, brainstem, and spinal cord (8). Involvement of the thalami and basal ganglia while infrequent is rather characteristic of ADEM (2). The extension of the lesions, particularly involvement of the brainstem has been associated with worse prognosis (9). As lesions may evolve during weeks, the pattern of enhancement may be inconsistent, with up to one-third of patients showing some lesions without associated enhancement (2, 10, 11).

ADEM is pathologically characterized by the perivenular infiltrates of T cells and macrophages associated with perivenular demyelination with limited sleeves of demyelination (12–14) unlike the confluent areas of demyelination seen in MS.

Clinical course of ADEM is considered in general to be favorable with reports of spontaneous improvement, although permanent disability and death have also been reported. Clinical outcome is in general more severe in adults compared to children (15). Impaired consciousness and occurrence of seizures have been associated with poor prognosis (16, 17). Complete motor recovery ranges between 15 and 46% in adults (2), 20% relapse within 2 years (15).

Initial management consists of high-dose IV corticosteroids followed by tapering with oral corticosteroids (18, 19). If no improvement is noticed after a course of corticosteroids is completed, other therapeutic options should be considered. Multiple reports and studies have reflected clinical improvement after administration of IVIG, especially when an infectious trigger is identified (20, 21). Clinical improvement after IVIG administration can be seen from the initial course up to 3 weeks (21, 22). Plasmapheresis has also been shown to improve outcome in randomized trials (23, 24). The clinical response after four to six sessions of plasmapheresis in refractory cases is usually seen after 3 days of starting therapy (25–27). These therapeutic options have been used individually or in combination (28). Other immunomodulatory agents as cyclophosphamide and rituximab have been used in the management of other fulminant demyelinating diseases (7, 29). Other non-pharmacological measures such as decompressive hemicraniectomy and hypothermia have reported effective in fulminant cases (30, 31). Currently, there are no data from prospective randomized trials for the management of ADEM in children or adults.

In our case, considering that the half-life of IVIG ranges between 22 and 96 days, the patient might not have had enough exposure to IVIG before it was removed from the system by the plasmapheresis, precluding him from receiving the full benefit of IVIG. However, given his deteriorating clinical course and that time was of the essence, starting plasmapheresis sooner was considered a sound clinical decision.

## Summary

We report an adult with fulminant presentation of ADEM with no clinical or radiological evidence of improvement despite multimodal aggressive parenteral therapy.

## Conflict of Interest Statement

The Specialty Chief Editor Gregory Gruener declares that, despite being affiliated to the same institution as the authors, the review process was handled objectively and no conflict of interest exists. The authors declare that the research was conducted in the absence of any commercial or financial relationships that could be construed as a potential conflict of interest.
